# Rickettsial seropositivity in Lao PDR smallholder livestock farms: Implications for animal and human health

**DOI:** 10.1016/j.onehlt.2026.101327

**Published:** 2026-01-10

**Authors:** Chantal Tawfik, James R. Young, Syseng Khounsy, Phouvong Phommachanh, Peter Christensen, Watthana Theppangna, Tom Hughes, Jantana Wongsantichon, Stuart D. Blacksell, Michael P. Ward

**Affiliations:** a Sydney School of Veterinary Science, The University of Sydney, Camden, NSW, Australia; b Mahidol Oxford Tropical Medicine Research Unit (MORU), Faculty of Tropical Medicine, Mahidol University, Bangkok, Thailand; c National Animal Health Laboratory, Vientiane, Laos; d Conservation Medicine, Sungai Buloh, Selangor, Malaysia; e Centre for Tropical Medicine and Global Health, Nuffield Department of Medicine, University of Oxford, Oxford, United Kingdom

**Keywords:** Rickettsia, Livestock, One health, Epidemiology, Hotspots, Laos

## Abstract

Rickettsioses are emerging zoonotic diseases in Southeast Asia caused by vector-borne, intracellular *Rickettsia* bacteria that threaten public health, animal welfare, and food security. Despite their importance to rural livelihoods, the epidemiology of rickettsial exposure in livestock remains poorly understood. This study used abattoir-based serology to evaluate livestock as sentinels of environmental exposure to Rickettsia in Lao PDR and to identify spatial hotspots and risk factors for seropositivity. Abattoir-based serological data were generated from cattle, pigs, and water buffalo samples collected in 18 provinces between January 2022 and April 2023. The immunofluorescence assay was used to detect antibodies against three rickettsial antigens: Scrub Typhus Group (*Orientia tsutsugamushi*), Typhus Group (*R. typhi*), and Spotted Fever Group (*R. conorii*, *R. honei*). Samples with IgG titres ≥1:100 were considered positive. Of 821 samples tested, 32 were seropositive: 25 cattle (9.9%), seven pigs (2.4%), and none of the buffalo. Breed and age were significant predictors, with non-native breeds and animals under one year old more likely to be positive (*p* < 0.05). Spatial-temporal analysis revealed one significant cluster in cattle (*p* = 0.0056) in southern Laos. These results represent the first nationwide serological assessment of Rickettsia exposure in Lao livestock.

## 1 Introduction

Rickettsial diseases are one of the leading causes of febrile illness in Southeast Asia (SEA), and a major cause of morbidity and mortality in humans [1,2]. They remain understudied, neglected, and often misdiagnosed, leading to inappropriate clinical management [3,4]. Rickettsial diseases are vector-borne, Gram-negative, intracellular bacteria in the order *Rickettsiales* [5]. Within this order, there are two main families: Anaplasmataceae and Rickettsiaceae. Anaplasmataceae includes the genera *Anaplasma*, *Ehrlichia*, and *Neorickettsia*, while the family Rickettsiaceae consists of the genera *Rickettsia* and *Orientia* [6]. The main antigenic rickettsial groups of human importance are the Scrub Typhus Group (STG), caused mainly by *Orientia tsutsugamushi*, the Spotted Fever Group (SFG) comprising more than 20 *Rickettsia* spp. and the Typhus Group (TG), which includes murine typhus (*Rickettsia typhi*) and epidemic typhus (*Rickettsia prowazekii*) [1,3]. These groups are categorised based on vectors, antigenicity and clinical manifestations [6].

Rickettsioses have many clinical manifestations depending on the subgroup and can vary from mild to life-threatening [6]. In humans, they generally cause fever, myalgia, lymphadenopathy, rash, respiratory distress, multi-organ dysfunction, and neurological deficits [3]. In domestic animals such as dogs, infection is most often asymptomatic, or if clinically affected, they present with non-specific signs including anorexia, depression, pyrexia and pale mucous membranes [7]. Although the presentation of rickettsioses in livestock varies, seropositive cattle rarely show clinical signs [9].

The Lao People's Democratic Republic (Lao PDR or Laos) is a landlocked country in Southeast Asia, located east of the Mekong River and bordered by Thailand, Myanmar, China, Vietnam, and Cambodia [8]. In Laos, more than 80% of the population lives in rural areas and works in the agricultural sector [8]. Whilst rickettsial prevalence and spatial distribution have been documented in humans in Laos and neighbouring countries [9], studies in livestock are limited. In previous studies, domestic animals have been found to have higher rickettsial infection rates in areas endemic for the disease than in non-endemic areas [10]. This suggests that domestic animals may reflect environmental exposure to rickettsial pathogens and, in some settings, act as amplifiers of infection risk, rather than true reservoirs. Similarly, studies have found that small mammal hosts, such as rodents, are seropositive for STG in Laos [11], suggesting a correlation between seropositive animals and increased human disease prevalence.

Livestock are considered amplifiers of some rickettsial pathogens, and identifying clusters of seropositivity might indicate areas of potential human disease risk [12,13]. Seroprevalence studies that use livestock as sentinels, therefore, offer a practical approach to identifying locations with heightened environmental exposure to Rickettsia. In this study, we aimed to identify spatial hotspots of rickettsial seroprevalence among smallholder livestock farms in Lao PDR and to assess whether factors such as species, age, sex, breed, and seasonality were associated with seropositivity across different locations. Identifying areas of high livestock seropositivity might help inform targeted surveillance and prioritisation of disease control resources.

## 2 Materials and methods

### 2.1 Ethics statement

The animal ethics for this study were approved by the Ministry of Agriculture and Forestry, Department of Livestock and Fisheries, Lao PDR; approval number 0019/DLF. The studies were conducted in accordance with the local legislation and institutional requirements. Written informed consent was obtained from the animal owners for the participation of their animals in this study.

### 2.2 Study design and sample collection

This was a cross-sectional, observational study, analysing serological data from January 2022 to April 2023. Each month, samples from 10 animals from cattle (*Bos taurus*), pigs (*Sus domesticus*) and water buffalo (*Bubalus bubalis*) were collected from abattoirs in 18 different provinces of Lao PDR (Attapue, Bokeo, Bolikhamxai, Champasak, Houaphan, Khammouan, Louangnamtha, Louangprabang, Oudomxay, Phongsaly, Salavan, Savannakhet, Vientiane CT, Vientiane PV, Xayaboury, Xaysomboun, Xekong, and Xiangkhouang). Trained government animal health workers collected serum via venipuncture, frozen, and stored at the National Animal Health Laboratory (NAHL) in Vientiane. A total of 7638 serum samples were collected between January 2022 and April 2023. Using a list of the serum bank samples, simple randomisation and systematic selection were used to select every 10th serum sample for rickettsial screening, with 18 animals of each species (pig, cattle, water buffalo) from the 18 provinces. No *a priori* sample size calculation was performed, as the number of samples tested was constrained by the availability of archived sera and diagnostic kit resources [14]. Samples with low volume or excessive red cell lysis were excluded. This left 821 samples for testing at the Mahidol Oxford Tropical Medicine Research Unit (MORU) in Bangkok, Thailand.

### 2.3 Serological analysis

Immunofluorescence assay (IFA) slides, produced by MORU [11], were used [11], coated with rickettsial antigens from STG (*O. tsutsugamushi*; Karp, Kato, Gilliam, and TA716 strains), TG (*R. typhi*), and SFG (*R. conorii* and *R. honei*). Serum samples were serially 2-fold diluted from 1:100 to 1:25600 in 2% skim milk in phosphate-buffered saline (PBS). Two μL of diluted serum were added to each well and incubated at 37 °C for 30 min. The wells were then washed 4 times with PBS, and 2 μL of FITC-conjugated anti-bovine (Invitrogen #A1–8752; 1:2000 dilution) or anti-pig (Invitrogen #61–9111; 1:3000 dilution) was added to each well. The slides were incubated and washed, a fluorescence mounting medium was added, and the slides were interpreted independently by two technicians. An IgG antibody-positive sample was defined as any with a titre of ≥1:100 in any of the rickettsial antigenic groups (i.e., TG, STG, or SFG). Antibody titres were reported as reciprocal dilutions from indirect immunofluorescence; values reported as <1:100 indicate titres below the assay cutoff. For analyses and figures, we treated titres numerically where explicitly recorded (i.e., 1:100 as 100, 1:200 as 200) while preserving <1:100 as censored values.

### 2.4 Spatial and statistical analysis

Microsoft Excel 2025 (Microsoft Excel for Mac Version 16.97 (25051114)) was used to analyse the data. The data record sheet included the individual animal number, sample ID, species name, STG IgG titre, TG IgG titre, SFG IgG titre, age, sex, point of origin (coordinates), date collected, vaccination status, and owner name. The data was cleaned, and samples with missing or illogical values were excluded. Spelling errors were identified and corrected, and formats were standardised. This data was categorised into tables (using the pivot table function in Excel) to further classify the seropositive samples by antigenic groups (TG, SFG and STG), species, age (≤ 1 year, 2–5 years and ≥ 6 years), sex (male and female), breed (exotic, native, and mixed), seasonality (dry season (November – April) and wet season (May – October). Pivot tables were used to collate this data.

The data were categorised into 101 unique locations based on the point of origin of each sample, using longitude (x) and latitude (y). Each unique location was considered a herd or a village. At each location, the total number of animals, total number of seropositive animals, total number of cattle, total number of seropositive cattle, total number of pigs, total number of seropositive pigs, total number of water buffalo, and total number of seropositive water buffalo were tabulated. This process was repeated for each province, resulting in provincial-level data being tabulated.

ArcGIS Pro 3.1 mapping software (ESRI, Redlands, CA, USA) was used to create a visual representation of the data. Proportional symbol maps were created by importing seroprevalence data and coordinates (Microsoft Excel) and displaying them on the Laos national shapefile (accessed from GADM version 2.8). Proportional symbol maps of total cattle sampled, positive cattle, total pigs sampled, and positive pigs were created. The provincial data were used to create choropleth seroprevalence maps using the Laos national shapefile (GADM version 2.8) via a spatial join based on province ID numbers. Three maps were created: a prevalence map of all animals sampled, and two species-specific maps demonstrating rickettsial seroprevalence in pigs and cattle.

Once these maps were created, global clustering of seropositivity was tested using Moran's spatial autocorrelation (Spatial Statistics, ESRI). Using the coordinates of sampled animals, Moran's index was calculated for seropositive animals (cattle and pigs) and for sampled animals (cattle and pigs). In this analysis, the data consisted of the X and Y coordinates and the number of samples or positives (Z) at each location. A Moran's index of 0 indicates that the number of samples or the number of positives at locations does not depend on location, i.e. spatial randomness. A positive index indicates that the number of samples (or number of positives) at locations closer together are more similar (clustering); a negative index indicates overdispersion. A *p*-value of *p* < 0.05 was used to assess the statistical significance of Moran's index.

Clusters (hotspots) of seropositivity were identified using a scan statistic (SaTScan v9.6). A Bernoulli model was used, with data consisting of seropositive (1) and seronegative (0) samples for both cattle and pigs. A scanning window of up to 50% of the study area was used. A Monte Carlo simulation (999) was used to estimate the *p*-values of the clusters. Statistically significant clusters were identified using *p* < 0.05 [14]. In addition to a spatial analysis, a spatial-temporal analysis was also undertaken. The spatial scanning window used was 50% of the study period.

The association between risk factors (sex, breed, age, species and seasonality) and serological status (positive, negative) was assessed using logistic regression models (Jamovi). Associations were interpreted using odds ratios and 95% confidence intervals. Variables associated with serostatus at *P* < 0.2 were included in a stepwise multivariate logistic regression model to determine the best set of predictors of Rickettsia serostatus. The best model was selected based on the likelihood statistic, and model fit was assessed using the Hosmer-Lemeshow statistic.

## 3 Results

### 3.1 Overall

Between January 2022 and April 2023, after applying the exclusion criteria, 821 serological samples were collected from abattoirs in 18 different provinces of Lao PDR (Attapue, Bokeo, Bolikhamxai, Champasak, Houaphan, Khammouan, Louangnamtha, Louangprabang, Oudomxay, Phongsaly, Salavan, Savannakhet, Vientiane CT, Vientiane PV, Xayaboury, Xaysomboun, Xekong, and Xiangkhouang). From the available sampling frame, 252 cattle, 286 pigs and 283 water buffalo serum samples were selected for testing. There were 32 seropositive animals in total, of which 25 were cattle, 7 were pigs, and there were no seropositive water buffalo samples (Table 1). Of the 32 seropositive animals, 97% (*n* = 31) tested positive for TG. All 25 seropositive cattle tested positive for TG, and one concurrently tested positive for STG. Of the seven seropositive pigs, six tested positive for TG, and four were also positive for SFG. One pig was only seropositive for SFG. All detected antibody titres were low (≤1:200), with most positive samples at or close to the assay cutoff (1:100).

**Table 1:** Seroprevalence of rickettsial subgroups (TG, STG and SFG) in smallholder livestock (cattle, pigs and water buffalo) in Lao PDR, 2022–2023, estimated from abattoir-based monitoring.

				Maximum observed IgG titre		
Species	Positive, any test	Total sampled	Prevalence (%)	STG IgG	TG IgG	SFG IgG
*B. taurus*	25	252	9.9	1:100	1:200	1:100
*S. domesticus*	7	286	2.4	1:100	1:200	1:100
*B. bubalis*	0	283	0	1:100	1:100	1:100
Total	32	821	3.9			

The maximum titres observed were: STG and SFG IgG 1:100 (all species) and TG IgG 1:200 (cattle and pigs) (Table 1).

### 3.2 Breed

Rickettsial seropositivity status was positively associated with breed (*p* < 0.001). Most seropositive cattle (*n* = 24) were mixed or exotic breeds. Multivariate stepwise logistic regression indicated that breed alone was the best predictor of seropositivity status. Exotic breeds were 640 times more likely to be seropositive than native breeds (odds ratio [OR] 640, 95% confidence interval [CI] 72–5666, *p* < 0.001). Similarly, mixed breeds were 59 times more likely to be seropositive than native breeds (OR 59, CI 8–441, p < 0.001) (Table 3).

### 3.3 Age

**Fig. 1:**
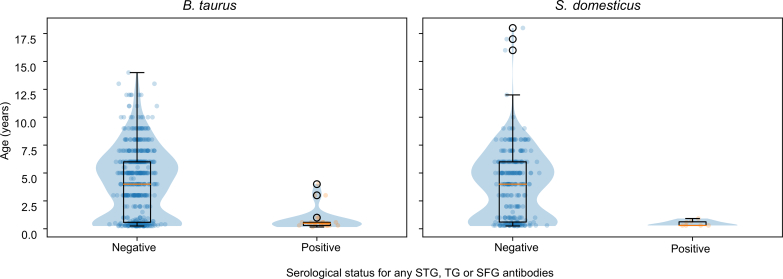
Age distribution by overall rickettsial serostatus and species. Violin plots show the density of age (years) among seronegative and seropositive animals within each species, overlaid with boxplots (median and interquartile range) and individual observations. Across species, seropositive animals were predominantly young, whereas seronegative animals spanned a wider age range.

Rickettsial seropositivity was positively associated with age (p < 0.001). All seropositive cattle were less than 4 years old, with 21 out of the 25 seropositive cattle being less than a year old. The highest seroprevalence was observed in animals younger than one year (10.7%), declining sharply in animals aged 1–4 years (1.1%) and with no seropositive animals detected among those aged ≥5 years (Fig. 1). Seropositive animals were predominantly young, whereas seronegative animals spanned a broad age range extending into older age classes. Antigen-specific age stratification was not performed due to the very small number of STG- and SFG-positive samples, which precluded meaningful age-based analysis. Animals >1 year old had significantly lower odds of seropositivity compared to animals ≤1 year old (OR 0.047, 95% CI 0.014–0.154, *p* < 0.001). There was no statistical difference in seropositivity between animals aged 2–5 years and those aged>6 years (Table 2).

**Table 2:** Univariable binomial regression analysis of risk factors for rickettsial seropositivity status in Lao PDR, 2022 to 2023.

Variable	Estimate^1^	SE	Odds ratio	Lower CI	Upper CI
Species					
*B. taurus* and *B. bubalis* (baseline: *S. domesticus*)	0.670	0.434	1.954	0.835	4.575
Age					
> 1 year (baseline: ≤ 1 year)	−3.070	0.611	0.047	0.014	0.154
Breed					
Exotic (baseline: Native)	6.460	1.110	640.1	72.3	5666.4
Mixed (baseline: Native)	4.080	1.030	59.0	7.9	440.8
Sex ⁎					
Male (baseline: Female)	−0.685	0.400	0.504	0.230	1.103
Seasonality					
Wet, May–October (baseline: Dry, November – April)	0.158	0.367	1.171	0.570	2.406

1 Estimates represent the log odds of seropositivity status positive versus seropositivity status negative.

⁎ Two samples excluded with missing sex data (*n* = 819).

### 3.4 Species, sex and seasonality

Species, sex and seasonality were not statistically significant predictors of seropositivity status (*p* > 0.05).

### 3.5 Geographic distribution

**Fig. 2:**
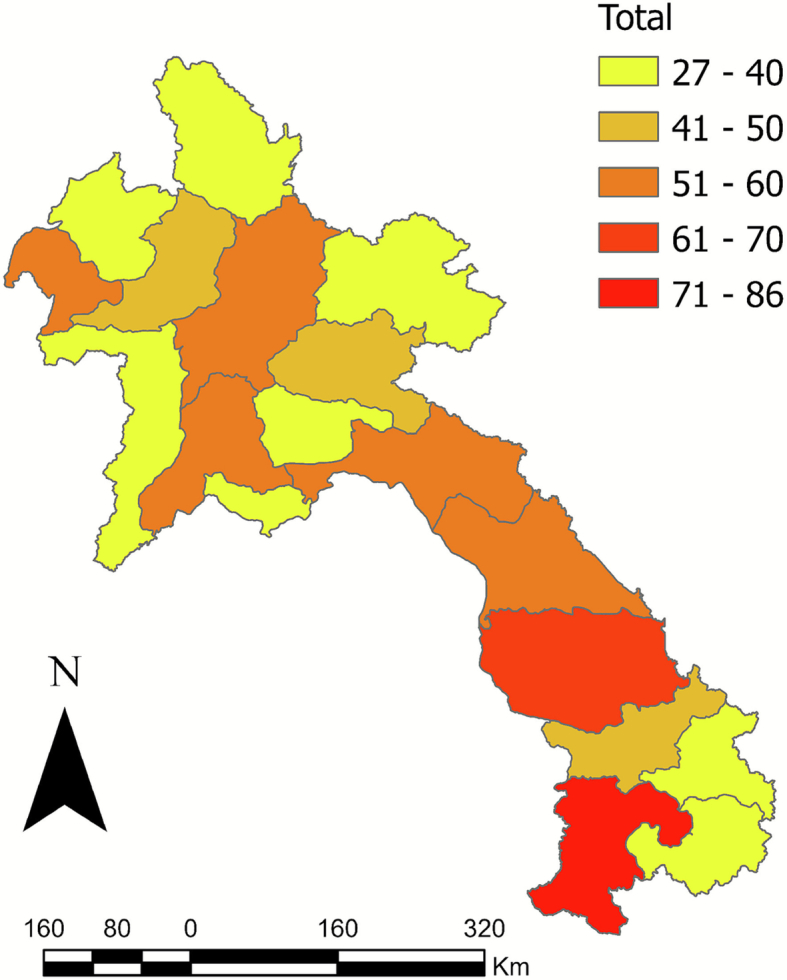
Geographical distribution of rickettsial sample size (TG, SFG and STG) among all the livestock species (cattle, pigs and water buffalo) tested in Lao PDR (2022−2023) using the MORU IFA. The scale bar and north arrow are included. Map shapefiles were sourced from https://diva-gis.org/data.html. Moran's global autocorrelation index was −0.1968 for total cattle (*p* = 0.218) and − 0.1867 for total pigs (*p* = 0.192).

**Fig. 3:**
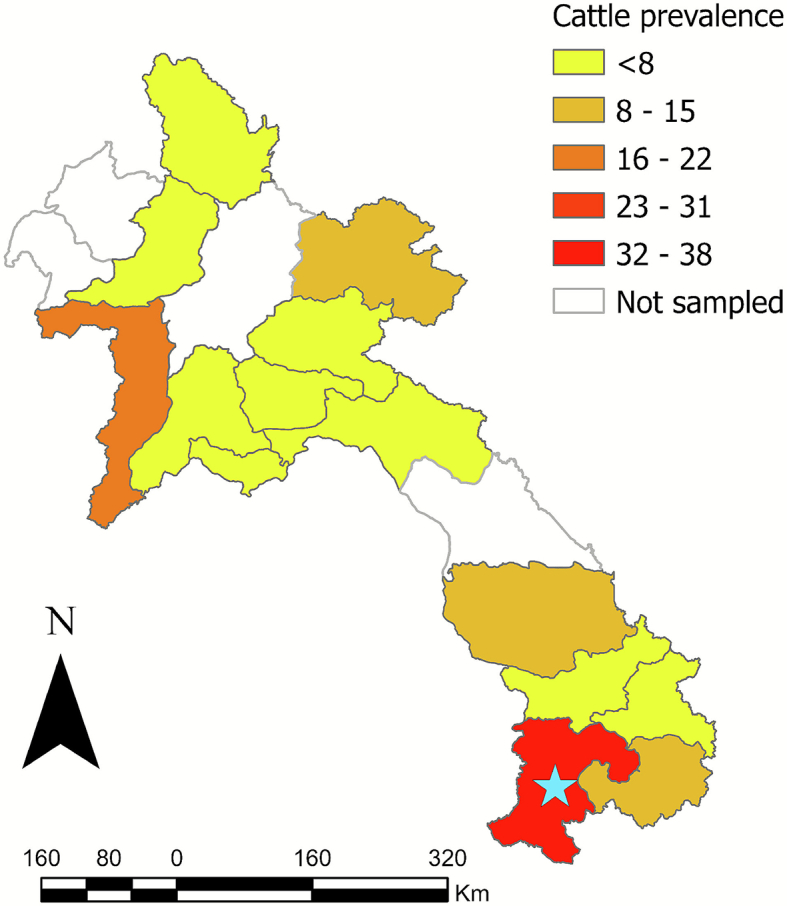
Geographical distribution of rickettsial seroprevalence (TG, SFG, and STG) among cattle tested in Lao PDR (2022–2023) using the MORU IFA (positive titre   ≥ 1:100). The blue star represents a significant cluster (p = 0.0056) at 14.7513 N 106.062 E with a radius of 0.49 km, via SaTScan v 9.6. The scale bar and north arrow are included. Map shapefiles were sourced from https://diva-gis.org/data.html. Moran's global autocorrelation index was 0.2881 (*p* = 0.265728).

**Fig. 4:**
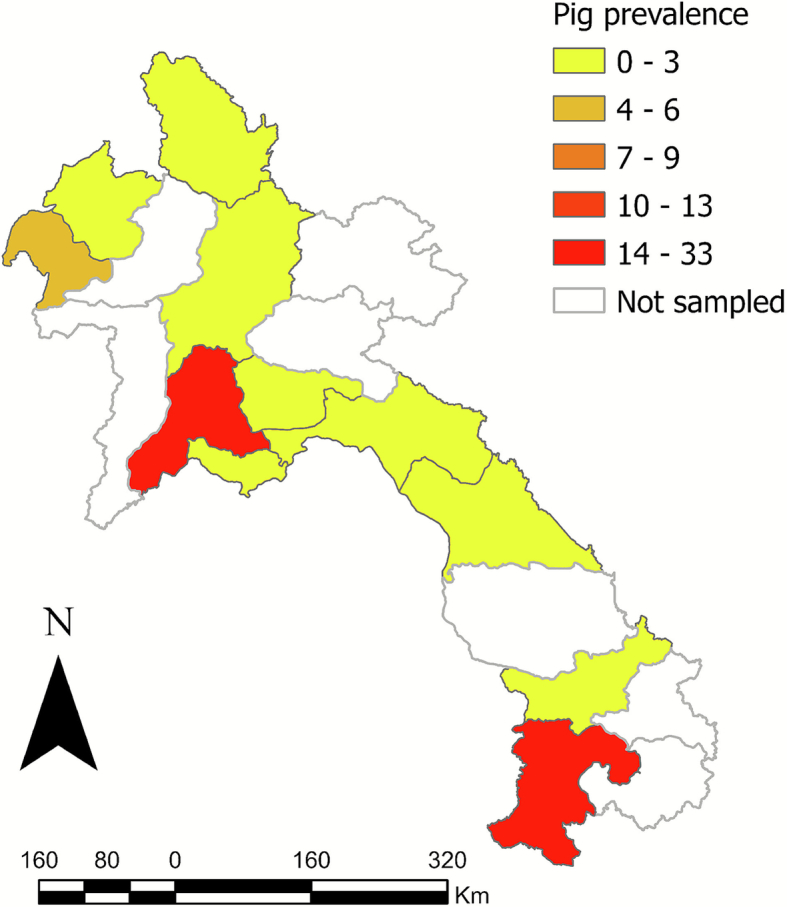
Geographical distribution of rickettsial seroprevalence (TG, SFG, and STG) among pigs tested in Lao PDR (2022–2023) using the MORU IFA (positive titre  ≥ 1:100). The scale bar and north arrow are included. Map shapefiles were sourced from https://diva-gis.org/data.html. Moran's global autocorrelation index was 0.0241 (*p* = 0.291422).

Of the 18 provinces sampled, Xayaboury had the highest total prevalence (16%) (Fig. 2). Champasak had the highest seroprevalence for cattle (38%) and pigs (33%) (Table 3). The spatial autocorrelation analysis (Global Moran's Index) for both total cattle sampled (−0.1968) and total pigs sampled (−0.1867) indicated that the data were over-dispersed, suggesting the dataset was likely geographically representative. The Global Moran's Index for positive cattle (0.2881) indicated global spatial clustering, although this was not statistically significant (*p* > 0.05) (Fig. 3); positive pigs (prevalence 0.0241 or 2.4%) were randomly spatially distributed (p > 0.05) (Fig. 4). The retrospective spatial cluster analysis (SaTScan) revealed a significant cold spot of positive samples (*p* = 0.0044) in central Laos (103.939 N,18.3477 E) with a radius of 2.07 km. The SatScan also revealed one marginally significant (*p* = 0.0056) time-space hotspot in cattle between 20/10/2022–20/03/2023 in southern Laos (106.062 N,14.7513 E) with a radius of 0.49 km (Fig. 2).

**Table 3:** Seroprevalence of Rickettsia by province in Lao PDR, 2022–2023, estimated from abattoir-based monitoring.

	*B. taurus*		*S. domesticus*		*B. bubalis*		Overall		
Province	Positive	Total	Positive	Total	Positive	Total	Positive	Total	% Prevalence
Attapue	4	39	0	0	0	0	4	39	10.3
Bokeo	0	0	2	51	0	0	2	51	3.9
Bolikhamxai	0	4	1	48	0	0	1	52	1.9
Champasak	5	13	2	6	0	67	7	86	8.1
Houaphan	4	27	0	0	0	0	4	27	14.8
Khammouan	0	0	0	1	0	54	0	55	0
Louangnamtha	0	0	0	27	0	1	0	28	0
Louangprabang	0	0	0	48	0	4	0	52	0
Oudomxay	0	8	0	0	0	36	0	44	0
Phongsaly	0	27	0	6	0	0	0	33	0
Salavan	0	5	0	40	0	3	0	48	0
Savannakhet	6	65	0	0	0	0	6	65	9.2
Vientiane CT	0	11	0	17	0	3	0	31	0
Vientiane PV	0	9	2	14	0	34	2	57	3.5
Xayaboury	6	37	0	0	0	0	6	37	16.2
Xaysomboun	0	1	0	28	0	2	0	31	0
Xekong	0	1	0	0	0	37	0	38	0
Xiangkhouang	0	5	0	0	0	42	0	47	0
Total	25	252	7	286	0	283	32	821	3.9

## 4 Discussion

In this study, we found that among livestock tested across the 18 Lao provinces, the prevalence of rickettsial seropositivity was low (<10%). We found that exotic and mixed breed and age (< 1 year) are significantly associated with rickettsial seropositivity. No seasonality was identified. Sex and species were not significant predictors of seropositivity, consistent with several studies in large ruminants, although effects have been reported in small ruminants in other settings. No water buffaloes were seropositive, consistent with findings that low haemoparasite prevalence may be associated with some level of resistance [15].

Limited diagnostic capacity and funding hinder awareness of vector-borne diseases such as Rickettsia [6,8]. Collaborative efforts, such as this project, aim to enhance laboratory capacity for emerging diseases, improving public health and disease response in Laos [6,16]. Abattoir surveillance identifies disease risk factors [17,18], but can be costly; targeting hotspots makes it more feasible, highlighting the need for further research. The data collected in this study were spatially over-dispersed, indicating the sampling was representative of the country. Our study sampled from abattoirs across all Lao provinces, making it more geographically representative. Abattoir sampling allows the collection of diagnostic material from various source animals, including those with subclinical disease, which is common in livestock [12]. It provides an efficient method for understanding disease epidemiology and detecting emerging diseases, enabling rapid responses and biosecurity measures.

This study revealed that only breed and age were significant predictors of rickettsial seropositivity, with breed as the strongest predictor: mixed and exotic breeds were more likely to be seropositive than native breeds. In animals aged ≤1 year, detected antibodies might partly reflect maternally derived (colostral) antibodies rather than direct exposure. Similar phenomena have been described in other mammalian species, and longitudinal studies would be required to distinguish passive antibody transfer from true seroconversion in livestock in Lao PDR. Alternatively, other studies suggest that maternal antibodies protect younger livestock and, as a result, they have a lower incidence of disease [19]. In other studies, authors have found that abattoir sampling is often biased toward younger animals, which could explain our higher rickettsial seroprevalence in young animals compared with other studies. [17].

Our study found no significant difference in seroprevalence between wet and dry seasons in Laos. [20] noted that cattle outbreaks of tick-borne disease are more likely in the wet season due to increased tick prevalence due to optimal environmental conditions. Another study found seropositivity in Laos varies by Rickettsia antigenic group: scrub typhus peaks in July and August, while murine typhus peaks in April and May [9]. In our study, TG was most prevalent among livestock (97%) with no significant seasonal variation. We identified a marginally significant cluster in cattle during the dry season (October–March), consistent with the literature highlighting TG's prominence during dry seasons [9]. This suggests cattle can serve as serological sentinels of environmental exposure to murine typhus.

Although this study did not directly investigate arthropod vectors, the rickettsial antigenic groups detected are associated with distinct transmission cycles. Typhus group rickettsiae are typically maintained in rodent-flea systems, spotted fever group rickettsiae in ticks, and scrub typhus group agents in chigger mites. Livestock seropositivity, therefore, likely reflects environmental exposure to infected vectors rather than reservoir competence, reinforcing their role as sentinels of local transmission ecology.

This study has several limitations. Our data lacks detailed information about the source, housing, and management of the animals, making it difficult to interpret results. A study on Sudanese livestock showed intensive housing reduces Rickettsia seroprevalence [19], possibly explaining higher rickettsial seroprevalence in cattle, as pigs are often housed intensively while cattle are mostly extensively housed [21]. Free-range cattle are more prone to parasitic infestations. Missing data on housing and management are common limitations in abattoir sampling; other studies also found that animal histories, vaccination, and origin data are often missing or inaccurate [17]. We assumed animals slaughtered in a province were raised there. Although the sampling was representative of the country and all provinces, only one livestock species was sampled in some provinces, indicating a need for more sampling per province. We lacked information on antemortem examination status, making it hard to interpret animal selection. If some animals might have shown signs of illness (e.g. poor condition, fever, weakness) at the time they were sent for slaughter, it could bias prevalence estimates; alternatively, poorly performing animals might be slaughtered elsewhere, leading to underestimation. Resource constraints limited the sample size, affecting disease monitoring and outbreak investigation, a common issue in Southeast Asia. The IFA, the serological gold standard for quantitative rickettsial antibody detection, has limitations, including operator subjectivity and cut-offs, and requires specialised equipment and expertise, reducing accessibility in rural areas [[22], [23], [24]]. Cross-reactions between antigenic groups (SFG and TG) can cause some animals to test positive for multiple groups. Rickettsial IgG indicates exposure, not current infection [25]. Animals might have been infected previously or lost IgG over time, complicating the relationship between serostatus and age.

In many Asian countries, tick-borne disease causes significant economic losses in the agricultural sector, primarily affecting marginal farmers on smallholder farms [20]. In the context of rickettsioses, farmers and agricultural workers account for most scrub typhus cases in countries such as China, Thailand, Vietnam, and Laos [9], highlighting contact with livestock and rickettsial exposure and infection among people [22]. Livestock may act as sentinels of environmental exposure and, in some contexts, as amplifiers of infection risk, rather than true reservoirs of rickettsial pathogens. Although clinical disease was not assessed, rickettsial exposure in livestock might indirectly affect animal welfare and productivity, especially where co-infections are common [12,13]. The close human–livestock relationship in Southeast Asia, combined with the zoonotic nature of rickettsial diseases, increases spillover risk for smallholder farmers [26]. These findings support the use of livestock as sentinels for identifying areas of increased rickettsial exposure risk and highlight the need for integrated One Health surveillance in Southeast Asia.

## Funding

The authors declare that financial support was received for the research, authorship, and/or publication of this article. The project or effort depicted was or is sponsored by the 10.13039/100000005Department of Defense, 10.13039/100000774Defense Threat Reduction Agency. The content of the information does not necessarily reflect the position or the policy of the federal government, and no official endorsement should be inferred [contract number HDTRA1–08-D-0007]. This research was funded in whole or in part by the 10.13039/100010269Wellcome Trust [220211/Z/20/Z] of the United Kingdom. For Open Access, the author has applied a CC-BY public copyright licence to any Author Accepted Manuscript version arising from this submission. Professor Michael Ward is the recipient of an Australian Research Council Australian Laureate Fellowship (FL240100037) funded by the Australian Government.

## CRediT authorship contribution statement

**Chantal Tawfik:** Writing – review & editing, Writing – original draft, Visualization, Formal analysis, Data curation. **James R. Young:** Writing – review & editing, Visualization, Methodology, Data curation, Conceptualization. **Syseng Khounsy:** Project administration, Investigation. **Phouvong Phommachanh:** Project administration. **Peter Christensen:** Project administration, Investigation. **Watthana Theppangna:** Project administration. **Tom Hughes:** Project administration. **Jantana Wongsantichon:** Resources, Project administration, Methodology, Investigation. **Stuart D. Blacksell:** Writing – review & editing, Visualization, Supervision, Resources, Project administration, Methodology, Funding acquisition, Conceptualization. **Michael P. Ward:** Writing – review & editing, Writing – original draft, Validation, Supervision, Resources, Project administration, Methodology, Formal analysis.

## Declaration of competing interest

Rickettsial seropositivity in Lao PDR smallholder livestock farms: Implications for animal and human health.

No competing interests are declared for the study reported. All funding sources for this study have been declared.

## Data Availability

All data generated or analysed during this study included in this publication are available at the Open Science Framework at https://osf.io/e2xka/overview?view_only=9a70896100ef4d7a99cb75a73e206fa8
